# Birth prevalence and initial treatment of Robin sequence in Germany: a prospective epidemiologic study

**DOI:** 10.1186/1750-1172-9-9

**Published:** 2014-01-17

**Authors:** Scarlet Vatlach, Christoph Maas, Christian F Poets

**Affiliations:** 1 Department of Neonatology, Tuebingen University Hospital, Calwerstr 7, Tuebingen 72076, Germany

**Keywords:** Obstructive sleep apnea, Upper airway obstruction, Retrognathia, Preepiglottic baton plate

## Abstract

**Background:**

We conducted a monthly epidemiological survey to determine the birth prevalence of Robin sequence (RS) and the use of various therapeutic approaches for it.

**Methods:**

Between August 2011 and July 2012, every pediatric department in Germany was asked to report new admissions of infants with RS to the Surveillance Unit for Rare Pediatric Diseases in Germany. RS was defined as retro- or micrognathia and at least one of the following: clinically evident upper airway obstruction including recessions, snoring or hypoxemia; glossoptosis; feeding difficulties; failure to thrive; cleft palate or RS-associated syndrome. Hospitals reporting a case were asked to return an anonymized questionnaire and discharge letter.

**Results:**

Of 96 cases reported, we received detailed information on 91. Of these, 82 were included; seven were duplicates and two erroneous reports. Given 662,712 live births in Germany in 2011, the birth prevalence was 12.4 per 100,000 live births. Therapeutic approaches applied included prone positioning in 50 infants, followed by functional therapy in 47. Conventional feeding plates were used in 34 infants and the preepiglottic baton plate (PEBP) in 19. Surgical therapy such as mandibular traction was applied in 2 infants, tracheotomy in 3.

**Conclusion:**

Compared to other cohort studies on RS, surgical procedures were relatively rarely used as an initial therapy for RS in Germany. This may be due to differences in phenotype or an underrecognition of upper airway obstruction in these infants.

## Introduction

In 1911, Shukowsky was the first to identify a small mandible as responsible for dyspnea and cyanosis in newborns [1]. In 1923, the French stomatologist Pierre Robin described infants with a hypoplastic mandible and glossoptosis resulting in upper airway obstruction with or without a cleft palate [2,3]; he later became the eponym for this condition. In about 50% of affected children, RS is not isolated but associated with other, mostly syndromic anomalies [4-6]. Incidence figures are scant, ranging from 1:8500 for the Liverpool area to 1:14,000 for Denmark [7,8]; there are no such data for Germany. One reason for the rareness of birth prevalence data for RS is probably that no consensus on diagnostic criteria exists for this disorder.

RS can lead to complications such as failure to thrive, hypoxemia or cor pulmonale. Although less distinctive forms may only become apparent some weeks after birth with snoring or obstructive sleep apnea (OSA), mandibular hypoplasia appears to be the main problem in RS, particularly if it is associated with the tongue being shifted backwards (glossoptosis), so that its base compresses the epiglottis. Glossoptosis may result in a narrow pharynx and life-threatening respiratory distress [4,9].

At present there is no consensus about either diagnosis or treatment, and procedures applied seem heterogeneous, but epidemiologic data about their use are missing. We performed a prospective epidemiologic study in Germany to determine 1) the birth prevalence of RS, 2) the distribution of various treatments applied and 3) their perceived effect on airway patency and growth.

## Methods and patients

As part of the Surveillance Unit for Rare Pediatric Conditions in Germany (ESPED), all pediatric departments (459 contact persons) received monthly reporting cards asking them about new admissions of infants with RS between August 2011 and July 2012. Reports on the mailing card prompted immediate mailing of an anonymized three-page questionnaire. Electronic reminders were sent to all non-responders of the full questionnaire. In cases of persistent missing return of the completed full questionnaire, additional telephone requests were performed contacting the local person responsible for the ESPED collaboration (for details see [10]). Captured were all infants receiving inpatient health care in a pediatric unit during their first year of life independent of the indication leading to hospitalization. Infants with mild expressions of RS never admitted to a pediatric unit during this period were not included. Inclusion criteria were retro-/micrognathia in patients between 0–12 month of age as suspected by the attending physician showing at least one of the following additional criteria: upper airway obstruction, including sub-/intercostal retractions, snoring or hypoxemia, glossoptosis, feeding difficulties, weight < 3^rd^ percentile at admission, cleft palate or RS-associated syndrome. Regular analyses of ESPED’s return rate for completed questionnaires consistently exceeds 93% [10,11].

Hospitals reporting a case received an anonymized three-page questionnaire from the ESPED study center, designed by our group and asking for basic demographic data and clinical symptoms related to RS, occurrence of craniofacial disorders in the family, time of diagnosis, diagnostic procedures and treatment received, complications, nutrition and growth (online supplement). An anonymized medical report was also asked for. Having collected these data, we excluded cases that were reported in duplicate or did not meet inclusion criteria. To determine the birth prevalence of RS, we used data from the National Bureau of Statistics on the number of births in Germany [12].

As this is an explorative study without a primary hypothesis, no sample size calculations were done. Descriptive statistics were applied to characterize the study population. For evaluating weight gain, standard deviation score (SDS) for weight was computed using the Microsoft Excel add-in LMS Growth (version 2.14; http://www.healthforallchildren.com/?product=lmsgrowth) and compared using non-parametric tests. The reference population for this program is the British 1990 growth reference fitted by maximum penalized likelihood [13,14].

A p-value <0.05 was considered statistically significant. Analyses were done with statistical software (SPSS, release 21.0 for Mac; Chicago, Illinois, USA).

The study protocol, including a parental consent waiver, was approved by the ethics committee of Tuebingen University Hospital.

## Results

Between August 2011 and July 2012, a total of 96 patients with RS were reported to the study center; detailed information was supplied via returned questionnaires in 91, yielding a response rate of 95%. Of these 91 cases, 82 could be verified, two were erroneous notifications, having a diagnosis other than RS, and 7 were duplicate cases. Given 662,712 live births in Germany in 2011 a birth prevalence of 12.4 per 100,000 live births results. Patient characteristics are shown in Table 1. Unfortunately, several respondents did not provide answers to all items of the questionnaire. Response rates for single items therefore ranged from 70 to 100%.

**Table 1:** Patient characteristics

**Total number**	**82**
Initial presentation	
Retrognathia	82 (100%; N = 82)
Breathing difficulties	63 (89%; N = 71)
Glossoptosis	47 (76%; N = 62)
Feeding/swallowing difficulties	68 (92%; N = 74)
Weight < 3^rd^ percentile at admission	20 (35%; N = 57)
Cleft palate	58 (85%; N = 68)
RS-associated syndrome	28 (44%; N = 63)
Female	44 (54%; N = 82)
Age at admission (mean in days)	12 (min 0; max 301)
Gestational age (mean in weeks)	38 (min 30; max 41)
Age at discharge (mean in days)	48 (min 2; max 306)
Weight at birth (mean in grams)	2966 (min 890; max 4360)
Weight at admission (mean in grams)	3149 (min 890; max 7065)
SDS for weight	-0.72
Weight at discharge (mean in grams)	3690 (min 2340; max 7330)
SDS for weight	-1.46; p < 0.05

N refers to the number of questionnaires with data provided.

Of the 82 infants with RS, information about associated syndromes was provided in 56: 28 were isolated RS and 28 had additional syndromic features (Table 2). An underlying genetic disorder was diagnosed either by the attending pediatrician or a geneticist, but only 50% of infants with RS were referred to a geneticist.

**Table 2:** Associated syndromes

Undefined syndrome	10
Franceschetti syndrome	3
Carey-Fineman-Ziter syndrome	2
Goldenhar syndrome	2
Catel-Manzke-Syndrom (Klinodaktylie II & RS)	1
CHARGE association	1
Hallerman-Streiff-Francois syndrome	1
Craniodysostosis syndrome	1
Craniosynostosis syndrome	1
partial trisomy 11 q	1
Phelan-Mc-Dermid syndrome	1
Progeria	1
Stickler syndrome	1
Trisomy 13 (Pätau syndrome)	1
VACTERL association	1

In 13 children, the malformation was observed prenatally, in 7 pregnancies a polyhydramnion had been noted and a family history of a craniofacial condition was found in 8 patients. A diagnosis of RS was made on the day of birth in 58 infants, in 9 during the first week of life, in five in the first month and in one case after the first month of age.

Forty-two children (55% of N = 76 with information supplied) experienced respiratory difficulties necessitating respiratory support.

In the first week after birth, 40 patients received nasogastric tube feeding, 16 were fed via a Habermann feeder and in 10 conventional bottle feeding was used.

Other performed procedures are shown in Table 3 and implemented therapeutic interventions in Table 4.

**Table 3:** Investigations performed; N refers to the number of questionnaires with data provided

**Polysomnography (sleep study)**	**22 (34%; N = 64)**
Obstructive apneas identified	22 (100%; N = 22)
Endoscopy	27 (40%; N = 67)
Orthodontic investigation	43 (59%; N = 73)
Oral and maxillofacial investigation	55 (78%; N = 71)
ENT investigation	26 (40%; N = 65)
Genetic consultation	36 (50%; N = 72)
Neuropediatric investigation	42 (62%; N = 68)

**Table 4:** Implemented therapies

**Infants with available information on implemented therapies**	76*
Prone positioning	50
Continuous positive airway pressure	26
Nasopharyngeal tube	11
Orthodontic therapy	
Feeding plate	34
Preepiglottic baton plate	19
Tracheotomy	3
Oral and maxillofacial surgical therapy	
Osteoplastic distraction	0
Mandibular traction	2
Glossopexie	0
Functional therapy (orofacial regulation therapy, e.g. Castillo Morales)	47

*For 61/76 infants, more than 1 intervention was reported.

Nutritional supply at discharge occurred in 26 children via nasogastric tube, in 21 via feeding with a regular nipple and in 12 with the Habermann feeder. Fifty-six children were able to feed independently at discharge.

Considering SDS for weight, there was a decrease from -0,72 at admission to -1,46 at discharge (p < 0.05).

## Discussion

This is the first epidemiological study on Robin sequence from Germany. The 82 cases of infants with RS reported here correspond to a birth prevalence of about 1:8080, which is in the upper range of that reported by others [7,8,15]. Nevertheless our data still might represent an underestimation of birth prevalence as potentially some mild cases of infants with RS were missed if they had never been hospitalized in pediatric units. Another study found a birth prevalence of 1:2500 for infants with nonsyndromic cleft palates, about one quarter of these representing infants with RS [16]. Further incidence data on the disorder are sparse as many other studies consist of (large) case series [17-19].

Prenatal detection of RS was rare. In 71% of recorded infants a suspicion of RS was raised on the day of birth suggesting that in a substantial proportion of infants initial diagnosis and implementation of adequate diagnostic evaluation could have been missed or at least delayed. This might result in a significantly enhanced risk of affected babies from severe, underestimated breathing difficulties due to unrecognized UAO in their first days of life. Hence improvement of the prenatal detection rate of RS and prenatal referral to a reference center is a substantial concern, at least in Germany. Additionally, higher awareness in midwives and obstetricians of characteristic symptoms and potential consequences of UAO related to the disorder could contribute to earlier recognition of leading symptoms of RS and might allow for a more timely surveillance and treatment of affected neonates. This could potentially lead to an alleviation of long-term consequences resulting from unrecognized hypoxia.

When designing a survey such as this, applying a correct definition is paramount. In RS, this is hampered by the fact that there is considerable variability regarding its definition. To minimize potential underreporting, we used a rather broad definition (retrognathia) plus one of the other symptoms/components listed in a recent survey on diagnostic criteria for RS [20,21]. Reassuringly, however, 89% of infants were reported do have respiratory distress and 85% a cleft palate, i.e. the classic hallmarks of RS. Glossoptosis, however, was reported in only 71%. In our experience as a referral center for RS, glossoptosis may not always be detected by pediatricians not familiar with it.

The distribution of syndromic vs. non-syndromic RS is in line with other studies, although some syndromic forms of RS may have been missed as only 50% were referred to a geneticist [4,5]. Particularly Stickler syndrome may have been missed, as its clinical features are often not immediately apparent after birth [22].

When comparing the diagnostic procedures applied, we realized that only 34% of respondents had used polysomnography, the gold standard for detecting OSA in children with RS [23-25]. This may have led to an underrecognition of sleep-related upper airway obstruction, thus misinforming subsequent therapy.

Regarding treatment, we noted that surgical interventions, frequently reported by others [26,27], are apparently only rarely performed in Germany. Only 3 children received tracheostomy and 2 were treated with mandibular traction. Treatments most commonly applied were prone positioning in 50/82 infants, followed by functional therapy through a speech therapist (e.g. Castillo Morales); for details see Table 4. Castillo-Morales therapy, originally developed for children with Down syndrome, involves stimulation of the orofacial musculature to help relieving upper airway obstruction (UAO) [28], but has never been systematically studied in RS. Prone positioning has been described in some case series as a sufficient treatment modality in 50 to 80% of RS infants [19,29], but its effectiveness has also not been proven objectively (e.g., by polysomnography). Furthermore, it is concerning that studies report a more than 10-fold increase in the risk of sudden infant death syndrome in healthy infants placed prone for sleep, making it questionable whether parents can safely be advised to place their baby with RS prone for sleep [25].

In our survey, the use of palatal plates was reported in 53 infants. These have been shown to resolve glossoptosis and airway obstruction [30], however, feeding problems may persist. Use of the preepiglottic baton plate (PEBP) was reported in 19 infants (by 10 centers); it is yet the only intervention for RS whose effectiveness has been tested in a randomized controlled trial [31].

At the time of admission, 40 infants were tube fed, this number was reduced to 26 by the time of hospital discharge; SDS for weight allows assessing weight gain objectively. When comparing SDS for weight at admission and discharge, we saw a decrease by 0.7 standard deviations. Thus, although many infants were discharged without nasogastric tube feeding, they apparently did not gain weight appropriately. Unfortunately, underlying reasons remain unclear. Considering the fact that UAO in the majority of infants was treated with prone positioning as the sole intervention one may speculate that high energy expenditure due to insufficiently treated breathing difficulties based on UAO could be an important source of faltering growth. Poor feeding resulting from swallowing and breathing difficulties might be another reason. In order to elucidate the influence of different factors leading to faltering growth, such as enhanced energy expenditure due to undertreated UAO, feeding difficulties or a potentially underlying genetic disorder, growth data during infancy under different treatment modalities should be evaluated. This could lead to a better appreciation of growth problems in infants with RS and in the long term to a reduction of poor growth and its long-term consequences in this population.

We received information on cases only some weeks or months after admission. Our data may thus be subject to recall bias. As questionnaires were not always completed throughout, there is lacking information for several items, the latter also potentially biasing our results. Also, we have no way to ascertain how many cases were missed. Notably outpatients never having required pediatric inpatient treatment or having died prior to transport to a monitoring unit (i.e., a pediatric department) might not have been captured. Thus, patients with mild characteristics of RS might have been missed. This is a limitation of our study. Unfortunately, there are no guidelines in Germany as to whether an infant with suspected RS has to be admitted to a hospital as an inpatient for further evaluation and initial treatment. On the other hand, it is reassuring that our birth prevalence data are in the upper range of what has been reported by others [7,8], and response rates with the ESPED surveillance system are high [10]. Finally, our definition for RS differed from that used by others [21,32], leading to uncertainty in comparing our results with published data on birth prevalence rates for RS.

## Conclusion

The birth prevalence of RS in Germany is about 1:8000 life births and surgical therapeutic options, dominating the international literature, do not seem to play a major role. Nonetheless, our data confirm that achieving normal weight gain remains a challenge, and we have no data to ascertain whether UAO, frequently encountered in these infants, was resolved by the therapies applied.

## Abbreviations

(RS): Robin sequence; (PEBP): Preepiglotic baton plate; (OSA): Obstructive sleep apnea; (ESPED): Surveillance unit for rare pediatric conditions in Germany; (SDS): Standard devation score; (UAO): Upper airway obstruction.

## Competing interests

The authors have indicated they have no financial relationships relevant to this article or any conflict of interest to disclose.

## Authors’ contributions

Dr. SV coordinated the data collection, analyzed the data and wrote the first draft of this manuscript, Dr. CM revised the manuscript and helped with data collection, and CFP initiated and supervised the study and revised the manuscript. All authors read and approved the final manuscript.
